# A retrospective mixed-methods evaluation of a national ORS and zinc scale-up program in Uganda between 2011 and 2016

**DOI:** 10.7189/jogh.09.010504

**Published:** 2019-06

**Authors:** Felix Lam, Damien Kirchhoffer, Dennis Mike Buluma, Lorraine Kabunga, Patricia N Wamala-Mucheri, Kate Schroder, Audrey Battu

**Affiliations:** 1 Clinton Health Access Initiative, Boston, Massachusetts, USA; 2 Clinton Health Access Initiative, Kampala, Uganda; 3 Abt Associates, Kampala, Uganda

## Abstract

**Background:**

In Uganda, diarrhoea was the third leading cause of mortality among children under the age of five in 2010. To address this issue, the Ministry of Health (MOH) formed a national Diarrhoea and Pneumonia Coordination Committee (DPCC) in 2011. One of the objectives of the DPCC for reducing diarrhoea mortality was to increase the use of oral rehydration salts (ORS) and zinc. This study aimed to describe and evaluate efforts by the DPCC to increase ORS and zinc coverage.

**Methods:**

We conducted a retrospective mixed-methods evaluation to describe the activities conducted under the DPCC and evaluate the extent to which the committee’s goal of increasing ORS and zinc use was achieved. We conducted secondary analysis using Uganda’s Demographic and Health Survey from 2011 and 2016, analyzed cross-sectional private medicine outlet surveys from 2014 and 2016, analyzed ORS and zinc distribution data from the Uganda National Medical Stores, and reviewed program documents from DPCC partners.

**Results:**

Nationally, the proportion of children under five with diarrhoea treated with ORS and zinc increased from 1% (95% confidence interval (CI) = 1%, 2%) in 2011 to 30% (95% CI = 27%, 32%) in 2016. Among private medicine outlets, the adjusted odds of having any zinc in-stock was 1.5 (95% CI = 1.14, 1.97) times higher in 2016 than in 2014, and the retail price for a complete treatment (2 ORS sachets and 10 zinc tablets) declined by $0.19 (95% CI = -0.31, -0.06), or 14%.

**Conclusions:**

Use of combined ORS and zinc for treatment of diarrhoea in children under five significantly increased in Uganda during the program period. The range of activities conducted by the various members of the DPCC likely contributed to the increase in the use of combined ORS and zinc.

In Uganda, diarrhoea is a major cause of morbidity and mortality among children under five. In 2010, the incidence of diarrhoea was 3.26 episodes per child per year, which led to an estimated 21 million episodes of diarrhoeal disease and 12 200 deaths among children under five [1]. This represented 8% of under-five deaths at the time. To address this issue, the Ugandan Ministry of Health (MOH) Child Health department formed a national Diarrhoea and Pneumonia Coordination Committee (DPCC) in November 2011. The DPCC was chaired by the MOH and included various government departments, health sector development partners with programs focused on diarrhoea and pneumonia control, and private businesses such as pharmaceutical manufacturers and distributors. The DPCC worked to develop a national strategy for tackling diarrhoea and pneumonia and to align resources among the government and partners to drive implementation of the national strategy. One specific objective of the DPCC was to increase the use of oral rehydration salts (ORS) and zinc to treat diarrhoea [2]. In 2011, the percent of children with diarrhoea receiving zinc was only 2% and 44% for ORS [3].

ORS, which prevents and treats dehydration due to diarrhoea, has long been a cornerstone of diarrhoea control programs. It is estimated that national scale-up of ORS may avert up to 93% of diarrhoeal deaths [4]. Clinical studies of supplemental zinc therapy have demonstrated that zinc can reduce the duration, intensity, and reoccurrence of diarrhoea, particularly in malnourished children [5-9]. In 2004, these studies led the World Health Organization (WHO) and the United Nations Children’s Fund (UNICEF) to issue a joint statement to recommend that zinc be added as an adjunct treatment with ORS for management of childhood diarrhoea [10]. The Uganda MOH revised their national clinical guidelines in 2010 to align with the WHO/UNICEF recommendations [11].

The ORS and zinc scale-up efforts of the DPCC have yet to be evaluated and documentation of the implementation activities and lessons learned are dispersed throughout various reports. In this study, we aim to undertake a review of the national ORS and zinc scale-up efforts under the DPCC and to evaluate the extent to which coverage of ORS and zinc and other key outcomes, such as availability and prices, have changed in Uganda between 2011 and 2016. Our main research questions are listed in **Table 1**.

**Table 1:** Study methods and research questions

Data source	Type of data	Question(s) addressed
Secondary analysis of Demographic and Health Survey	Quantitative	Among children seeking care from public and private sources of care, did coverage of ORS and zinc significantly increase between 2011 and 2016?
Document review	Qualitative	What was the scope and scale of interventions implemented for increasing the use of ORS and zinc?
NMS ORS and zinc distribution	Quantitative	Did public sector distribution volumes of ORS and zinc treatment courses increase between 2011-2016?
Private wholesale distributor sales	Quantitative	Did private sales volumes of ORS and zinc treatment courses increase between 2011-2016?
Private medicine outlet surveys	Quantitative	Did private sector availability of ORS and zinc increase between 2014 and 2016?
		Did the price for ORS and zinc reduce between 2014 and 2016?

NMS – National Medical Store; ORS – Oral rehydration salts

## METHODS

We used a retrospective mixed-methods study design to describe and evaluate the activities under the DPCC. The intent for using a mixed-methods study design was to provide a comprehensive description of the scale and scope of activities undertaken by multiple partners of the DPCC and evaluate several of these components. We conducted a secondary analysis of the Uganda Demographic and Health Survey 2011 and 2016 to evaluate the change in ORS and zinc coverage as reported by caregivers of children under five and disaggregated the results by source of care (eg, public or private). To evaluate whether program activities could explain these changes, we collected and reviewed government reports and program documents from partners supporting the DPCC, and we summarized the activities undertaken. We also collected and analyzed ORS and zinc public sector distribution data from the Uganda National Medical Stores and private sales data from six wholesale distributors in Uganda. Lastly, we conducted cross-sectional surveys with private medicine outlets in 2014 and 2016 to evaluate changes in the availability and price of ORS and zinc. **Table 1** summarizes the methods used in our mixed-methods evaluation study and the specific purpose of each method. In the following sections, we describe the details for data collection and analysis for each method.

### Secondary analysis of Demographic and Health Survey

We conducted secondary analysis of Demographic and Health Survey (DHS) data from 2011 and 2016. We extracted variables related to diarrhoea case management for children who had diarrhoea in the two weeks prior to the survey and sampling design variables specific to each survey. The DHS data sets have variables on specific locations for where care or advice was sought for the diarrhoea illness. We generated binary variables for cases that sought care or advice from public sector sources (eg, government hospital, health center, or community health worker) and private sector sources (eg, private hospital, doctor, pharmacy, or shop). We appended the data sets together and evaluated whether the proportion of children with diarrhoea in the last two weeks prior to the survey received ORS and zinc changed significantly between survey periods and explored these changes by sources of care. The analysis was conducted using Stata 14 (StataCorp, College Station TX, USA).

### Document review

We reviewed country policies, guidelines, gray literature, reports, presentations, and policy briefs that we were able to find on the Internet and from contacts at the MOH and the main health development partners that were active in the DPCC. The following websites were searched for documents pertaining to ORS, zinc, and interventions to improve diarrhoea treatment coverage and quality in Uganda: the Uganda Ministry of Health’s online library, UNICEF Research and Reports, SHOPS Plus Resource Center, CCM Central, and the websites of the main partners involved in the DPCC. The search was restricted to English language publications with a publication date between January 1, 2007 and October 5, 2018.

We reviewed the documents for information specific to the description of activities undertaken and the organizational body responsible for the activities. We grouped data gathered from the document review into three categories: the Government of Uganda, health sector development partners, and private pharmaceutical companies. The three categories were decided upon based on *a priori* knowledge on the type of partners participating in the DPCC. Table S1 in **Online Supplementary Document** lists the partners who participated in the DPCC.

### ORS and zinc distribution and sales volumes

We worked with the Uganda National Medical Stores (NMS), an autonomous state-owned organization in charge of procuring, warehousing, and distributing medicines and health supplies to all public health facilities, to compile data on the monthly distribution of ORS, zinc, and co-packaged ORS and zinc for 2011 and 2016. We aggregated NMS distribution data by year using Microsoft Excel (Microsoft Inc., Seattle WA, USA).

In 2015, we also collected quarterly ORS and zinc sales volume data from six private manufacturers and distributors in Uganda. These private companies were selected since they were partners in the national effort and accounted for an estimated 90% or more of the ORS, zinc, and co-packaged ORS and zinc private market regarding volumes sold. We aggregated the sales data using Microsoft Excel (Microsoft Inc., Seattle WA, USA).

### Private medicine outlet surveys

We conducted cross-sectional private medicine outlet surveys in 2014 and 2016. The outlet surveys were conducted with private clinics, drug shops, pharmacies, and not-for-profit and religious clinics. There is no sampling frame of private outlets in Uganda, and so we adopted a multi-stage cluster sampling methodology used in other medicine audit studies conducted in Africa [12,13]. We used the 2001 Uganda national census as the sampling frame and randomly selected 168 census enumeration areas (EAs) with probability proportional to size. Trained enumerators visited the EAs and asked community members living in the EA to name all private sources of medicines that they patronize when their child is sick. From the list provided by the community, the trained enumerators visited each private outlet, explained the objective of the study, enrolled them in the study, and conducted a structured questionnaire with a respondent who works at the private outlet. The same EAs were used in both 2014 and 2016 and a complete listing exercise was conducted at each survey period.

n 2004, data collection was conducted by the Coalition for Health Promotion and Social Development (HEPS), a local, independent research agency. In 2016, we directly hired data collectors with previous experience conducting provider surveys as HEPS was already involved in another project. In both survey periods, the data collectors were trained for six days on the study objective, the listing protocol and tools, review of the questionnaire, use of the electronic data collection tool SurveyCTO (Dobility Inc, Cambridge MA, USA), research ethics, and a day of field practice. Data collection in 2014 spanned from October 16 to December 5, and data collection in 2016 occurred from March 14 to April 15. The questionnaire included questions on the private outlet’s characteristics, such as outlet type, years in business, and number of staff, and a physical audit of ORS, zinc, and co-packaged ORS and zinc at the outlet at the time of the visit.

For the analysis, we evaluated whether there was a change between 2014 and 2016 in the proportion of outlets with ORS and zinc in-stock on the day of the survey and whether there was a change in the mean price of ORS and zinc in Uganda. For availability of ORS and zinc, we defined seven outcomes: (1) whether both ORS and zinc were in-stock at the outlet on the day of the survey, either as a co-pack or individually packaged ORS and zinc; (2) any ORS in-stock, including as a co-pack or individually packaged sachets; (3) any zinc in-stock, including as a co-pack or individually packaged zinc tablets or syrups; (4) co-pack in-stock; (5) individually packaged ORS in-stock; (6) individually packaged zinc tablets in-stock; and (7) individually packaged zinc syrup in-stock.

Price was captured only if the outlet had the product in-stock on the day of the survey. We collected prices separately for each brand stocked at the outlet (if there was more than one brand), and we calculated the mean price of ORS and zinc by pooling prices across all outlets and brands. Among outlets with both individually packaged ORS sachets and individually packaged zinc tablets in-stock on the day of the survey, we calculated the price of purchasing two ORS sachets and 10 zinc tablets as this is considered a full treatment course and is equivalent to a co-pack in Uganda. For outlets stocking multiple ORS and zinc brands, we first calculated the average price for an ORS sachet and 10 zinc tablets across the brands in-stock at the outlet before calculating the total cost for a full treatment course. Prices were collected in Ugandan shillings (UGX). Prices collected in 2016 were adjusted for inflation to be comparable to 2014 using the ratio of the average national consumer price index for 2014 to the national average consumer price index for 2016 (Ratio = 1.12) [14]. We then converted the adjusted 2014 prices in UGX to 2014 US dollars (US$) during data analysis using the official mid-rate exchange as reported by the Bank of Uganda (UGX2600 per US$1) [15].

We appended the surveys from each period into a single data set. For the outcome of availability of ORS and zinc, we constructed logistic regression models to examine the association between the survey round and the odds of an outlet stocking ORS and zinc. For the outcome of price, we used ordinary least squares regression models to examine the association between the survey round and the mean retail price of ORS and zinc. For all models, we included outlet characteristic variables to control for potential confounders. The variables include the type of outlet (drug shop, pharmacy, private clinic, not-for-profit or religious clinic), region where the outlet was located (Central, Eastern, Northern, Western), whether the outlet was located in a rural area, the number of people working at the outlet, the number of years the outlet has been in business, the number of days in a week the outlet is open, and the number of hours in a day the outlet is open. The analysis was conducted using Stata 14 (StataCorp, College Station TX, USA).

Ethical approval for the outlet survey was obtained from Uganda National Council for Science and Technology (registration number: HS1562).

## RESULTS

### ORS and zinc coverage from secondary analysis of DHS

Secondary analysis of the DHS results are presented in **Table 2**. For ORS and zinc coverage, we found a statistically significant increase in coverage of combined ORS and zinc: from 1% (95% confidence interval (CI) = 1%, 2%) in 2011 to 30% (95% CI = 27%, 32%) in 2016. ORS coverage increased from 44% (95% CI = 40%, 47%) to 47% (95% CI = 44%, 49%) though the change was not statistically significant (*P* = 0.22).

**Table 2:** Proportion (95% confidence interval) of children with diarrhoea in the last two weeks that received ORS and combined ORS and zinc by survey and source of care

	DHS 2011	DHS 2016	*P*-value
**National**	N = 1684	N = 2923	
ORS	44% (40%, 47%)	47% (44%, 49%)	0.22
ORS and zinc	1% (1%, 2%)	30% (27%, 32%)	<0.01
**Sought care from public source**	N = 559	N = 949	
ORS	67% (62%, 72%)	77% (74%, 80%)	<0.01
ORS and zinc	2% (1%, 4%)	53% (49%, 57%)	<0.01
**Sought care from private source**	N = 730	N = 1151	
ORS	45% (40%, 50%)	50% (46%, 53%)	0.19
ORS and zinc	1% (1%, 2%)	33% (29%, 36%)	<0.01

DHS – Demographic and Health Survey, ORS – Oral rehydration salts

Among children who sought advice or care from a public source, we found a statistically significant increase in ORS coverage (*P* = 0.01) and combined ORS and zinc coverage (*P* < 0.01). ORS coverage increased from 67% (95% CI = 62%, 72%) to 77% (95% CI = 74%, 80%) and combined ORS and zinc coverage increased from 2% (95% CI = 1%, 4%) to 53% (95% CI = 49%, 57%). Among children who sought advice or care from a private source, we only found a statistically significant increase for combined ORS and zinc coverage (*P* < 0.01) – from 1% (95% CI = 1%, 2%) to 33% (95% CI = 29%, 36%) but not in ORS coverage (*P* = 0.19) – from 45% (95% CI = 40%, 50%) to 50% (95% CI = 46%, 53%).

### Document review

We found and reviewed 29 documents obtained through the search. The documents reviewed are listed in Table S2 in **Online Supplementary Document**. In the sections below, we describe our analysis of the documents pertaining to coordinated interventions conducted by the Government of Uganda, health development partners, and private suppliers between 2011 and 2016 to scale-up the use of ORS and zinc.

### Activities by the Government of Uganda

The Government of Uganda produced several health sector strategy documents that featured diarrhoea case management with ORS and zinc as a key component. The Child Survival Strategy in 2010, the Reproductive Maternal Neonatal Child Health Sharpened Plan for Uganda in 2013, and the Protect, Prevent, and Treat Implementation Plan in 2014, recommended approaches and activities to increase the availability and use of ORS and zinc by caregivers and health providers, such as focusing geographically in the regions and districts with the highest number of deaths, improving quantification and forecasting of health commodities at health facilities, and implementing Behavior Change Communication (BCC) activities to increase patient demand for ORS and zinc especially in the private sector [16-18]. The national strategies also set specific coverage targets for ORS and zinc. By 2017, the Government aimed for 90% of diarrhoea cases to receive Oral Rehydration Therapy (ORT), which includes ORS prepared from powder sachets, recommended homemade fluids, and/or increased fluids, and 30% to receive zinc.

In addition, the Government of Uganda updated several key policies to reflect the latest recommendations by the World Health Organization for the treatment of childhood diarrhoea. Specifically, the government added zinc to the national treatment guidelines for diarrhoea in 2010 and the national Essential Medicines List in 2012 [11,19]. During the same time period, it also adopted a notable policy to allow zinc to be given as an over-the-counter medicine beginning in 2013 [20]. While ORS could already be given without a prescription by pharmacies and drug shops, access to zinc had previously been restricted to only patients with a prescription. The new policy allowed zinc to be dispensed without a medical prescription and to be directly advertised to patients.

Following these updates to the national guidelines and strategy, NMS issued a tender to purchase ORS and zinc as a co-packaged product [20]. Prior to 2013, NMS procured and distributed ORS and limited zinc quantities, separately. Starting in September 2013, NMS introduced an ORS and zinc co-pack in all public health facilities.

**Figure 1:**
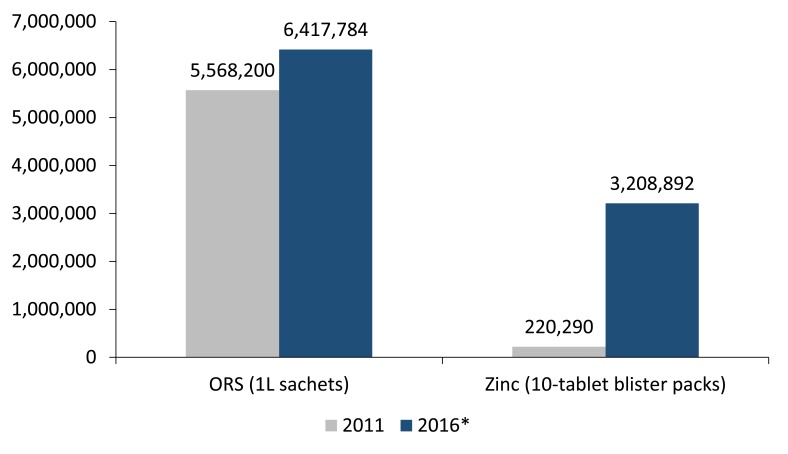
Number of ORS and zinc distributed by the National Medical Store (NMS) to public health facilities and community health workers. ORS – oral rehydration salts. *In 2016, NMS distributed 3 208 892 co-packs containing two 1L ORS sachets and a 10-tablet zinc blister pack in each co-pack.

**Figure 1** shows the distribution volumes for 2011 and 2016 by NMS to public health facilities. In 2011, NMS distributed 5 568 200 ORS sachets and 220 290 zinc blister packs to public facilities. By 2016, NMS no longer distributed individual ORS sachets and zinc blister packs, but distributed 3 208 892 co-packs. Since each co-pack has two sachets of ORS, the equivalent number of ORS sachets distributed by NMS in 2016 was 6 417 784 sachets, a 15% increase over 2011. NMS also increased their distribution volume for zinc by 1357%.

To expand access to vital diagnosis and treatment of childhood illnesses at the community level, the Government of Uganda introduced the integrated community case management (iCCM) program in 2010 [21]. The iCCM program trained community health workers, called village health teams (VHTs), to provide timely treatment to children with malaria, pneumonia, and diarrhoea at the community level [22]. For diarrhoea, the iCCM program trained VHTs to provide ORS and zinc to children with diarrhoea. Severe cases were referred to the nearest health facility. In addition, the VHTs conducted postnatal home visits in the first week after delivery, identified and referred sick newborns to the nearest health facility. In 2013, iCCM was implemented in 34 out of 112 districts by several health partners [21]. With funding from the Global Fund to Fight AIDS, Tuberculosis, and Malaria (GFATM), UNICEF and the President’s Malaria Initiative (PMI), the iCCM program was scaled up to 35 additional districts starting between 2015 and 2016, raising the total geographic coverage to 69 districts out of 112 [23]. The responsibility for distributing iCCM commodities, including ORS and zinc, to community health workers in the iCCM districts was transferred to NMS in September 2015. As NMS was already responsible for distribution to public health facilities, consolidation of iCCM commodities under NMS aimed to streamline and simplify quantification, warehousing, and distribution with the ultimate goal of reducing stock-outs at the community level [24].

Another key activity conducted by the Government of Uganda was engagement with the private manufacturers and suppliers regarding price regulation. Following a stakeholder consultation mediated by the DPCC in September 2013, the MOH and the National Drug Authority (NDA) endorsed the implementation of a Recommended Retail Price (RRP) in the private sector to help ensure affordability of ORS and zinc to patients purchasing ORS and zinc from private pharmacies and drug shops [25]. The RRP for one full treatment (two sachets of ORS and 10 tablets of zinc) was set at UGX1500 (US$0.58 using 2014 exchange rates) and communicated broadly to district health authorities, pharmacy wholesalers, private drug outlets, and consumers between October 2014 and end of 2015 [15,26].

### Activities by health development partners

The document review also revealed that several health development partners provided supporting activities that aligned with the priorities of the DPCC. Activities included interventions to ensure widespread availability and affordability of ORS and zinc products, strengthening diagnosis and treatment practices of public and private providers, and generating demand among caregivers of children [20,27,28]. A particular focus was placed on improving availability of ORS and zinc and dispensing practices among private providers. According to the DHS 2011, more than half of the children suffering from diarrhoea first sought care in the private sector [3].

Between 2013 and 2016, Abt Associates through the Strengthening Health Outcomes through the Private Sector (SHOPS) Project and the Clinton Health Access Initiative (CHAI) implemented several large-scale activities to improve availability and use of ORS and zinc among private health providers. In 2013, the SHOPS Project and CHAI, in partnership with the NDA and the Pharmaceutical Society of Uganda (PSU), trained more than 12 000 private providers on diarrhoea management across 97 districts of the 112 Ugandan districts [29]. During the first half of 2014, the SHOPS Project and CHAI deployed a team of 50 “ORS-zinc promoters” to approximately 150 wholesale pharmacies in the main trading centers across Uganda to educate drug retailers on the benefits of ORS and zinc when they visited the wholesalers and to encourage them to stock their shops with the treatments [29,30]. Between mid-2014 and the end of 2015, the SHOPS Project and CHAI deployed a team of 60 promoters to drug shops and private clinics in all the regions of Uganda except Karamoja and Kampala. The promoters conducted multiple visits to more than 16 000 unique drug shops (approximately 75% of all drug shops in Uganda) and approximately 3500 unique private clinics (about 75% of all private clinics in Uganda). The objectives of the visits to drug shops and clinics were: (1) to educate providers on the seriousness of childhood diarrhoea and the clinical benefits of ORS and zinc; (2) to share generic ORS and zinc marketing materials such as posters; (3) to link providers to affordable, quality wholesale suppliers in their area; and (4) to communicate the recommended retail price of ORS and zinc.

In addition to activities aimed to increase use of ORS and zinc among private providers, health development partners also supported the DPCC by implementing activities to improve the knowledge and health-seeking behaviors of caregivers of children under five. In 2012, antibiotics and antidiarrhoeals were the most requested treatments by caregivers [31]. Several health development partners used mass media and interpersonal communication to increase demand for and correct use of zinc and ORS. Radio was one of the mass media channels used to disseminate these messages. According to the DHS 2011, 65% of rural household had a radio, 54% of rural women listened to radio once a day, and 73% of rural women listened to radio at least once a week [3]. Between 2008 and 2013, the Uganda Health Marketing Group (UHMG) used regional television channels and radio stations to promote its own branded ORS and zinc [32]. Between 2014 and 2015, CHAI rolled out a national radio campaign that encouraged prompt care-seeking and stressed the benefits of ORS and zinc without mentioning any particular brand [33]. From the last quarter of 2014 onward, the campaign also communicated the Recommended Retail Price (RRP) for ORS and zinc sold in the private sector. The campaign aired a total of approximately 50 000 spot ads in four separate waves using up to 33 regional radio stations. Approximately 25 spot ads were aired per day on each participating radio station [34].

### Activities by the private pharmaceutical sector

In 2010, approximately 90% of the medicines consumed in Uganda were imported, mainly from India and China, and 10% were locally produced by 11 manufacturers [35]. Medicines are imported and distributed to drug outlets and clinics across the country through a network of about 10 first-line importers and approximately 400 Kampala-based and regional pharmacy wholesalers. Of the 11 local manufacturers, only one – Medipharm Industries Ltd – was engaged in the commercial production of ORS before 2011 [35]. As part of a United States Agency for International Development (USAID)-funded social marketing program, UHMG introduced one ORS product (RestORS) and the first zinc tablet formulation (Zinkid) in the private market in 2008 [36].

The DPCC and health development partners played a role in encouraging new product registrations. The number of ORS and zinc products registered and launched in the private market increased between 2011 and 2016. In 2011, there were two ORS products registered with the NDA – one zinc tablet product and no co-packaged product [37]. By 2016, there were six low-osmolarity ORS products – five zinc products and two co-packaged ORS and zinc products registered with the NDA [38]. **Table 3** presents the number of registered ORS and zinc products with the NDA in 2011 and 2016. A private supplier forum was established in 2013 to share ORS and zinc market intelligence data, behavior change communication investment plans, harmonize marketing messages and update private pharmaceutical companies about regulatory changes [39-42]. CHAI also provided technical support to local manufacturers to source affordable, quality pharmaceutical ingredients for producing ORS and zinc, registering their products with the NDA, and commercial sales and distribution strategy [33]. The wholesale cost of newly registered zinc products were 59%-82% lower than existing brands on the market [37].

**Table 3:** Number of ORS and zinc products registered with the National Drug Authority in 2011 and 2016

Product	2011	2016
ORS	2	6
Zinc tablet	1	5
Co-pack	0	2

In addition to the demand generation activities implemented by health development partners, several private suppliers used a range of sales and marketing techniques to increase the uptake of their brands of ORS and zinc, including the use of sales representatives, point-of-sales materials, and time-limited free sample distribution. In 2014, CHAI established formal partnerships with the two largest pharmaceutical distributors in Uganda. These partnerships included a quality assurance clause, a commitment by the distributors to maintain low ORS and zinc wholesale prices through a maximum resale price clause, volume and distribution reach targets, co-investment in marketing activities and modest financial incentives based on specific pre-defined volume, distribution reach, and price targets [30].

In 2012, UHMG sold 799 030 zinc 10-tablet packs, and at that time, they were the only distributors of zinc tablets in Uganda [36]. In 2015, six private wholesale distributors of zinc in Uganda (including UHMG) sold a total of 1 875 023 zinc 10-tablet packs, representing an increase of 135% over 3 years. In 2015, a total of 10 063 121 ORS sachets and 81 724 ORS and zinc co-packs were sold by the six private wholesale distributors.

### Private outlet surveys

We found 594 private outlets in 2014 and 699 in 2016 during the listing exercise. Of those, interviews were completed with 538 private outlets in 2014 and 588 in 2016. The remaining respondents declined to participate in the surveys. **Table 4** presents characteristics of the outlets at each survey period. There were some significant differences in the samples collected in 2014 and 2016. A greater proportion of the outlets in 2016 were from the Central region and urban areas, while a lesser proportion were from the Western region. The outlet sample in 2016 also had on average less staff and number of years operating as a business.

**Table 4:** Outlet characteristics (proportion, 95% confidence interval)

Outlet characteristics	2014	2016	*P*-value
	N = 538	N = 588	
**Outlet type:**			
Drug shop	0.65 (0.61, 0.70)	0.65 (0.59, 0.70)	0.80
Pharmacy	0.04 (0.03, 0.06)	0.03 (0.02, 0.06)	0.45
Private clinic	0.27 (0.23, 0.31)	0.31 (0.27, 0.37)	0.06
Not-for-profit clinic	0.03 (0.02, 0.05)	0.00 (0.00, 0.01)	<0.01
**Region:**			
Central	0.39 (0.35, 0.43)	0.47 (0.41, 0.54)	0.01
Eastern	0.23 (0.20, 0.26)	0.20 (0.17, 0.24)	0.22
Northern	0.13 (0.11, 0.17)	0.13 (0.10, 0.18)	0.97
Western	0.25 (0.21, 0.29)	0.19 (0.14, 0.25)	0.03
Rural	0.71 (0.67, 0.75)	0.61 (0.54, 0.67)	<0.01
Average number of people working there	3.1 (2.6, 3.5)	2.4 (2.1, 2.6)	<0.01
Average years in business	6.4 (5.7, 7.1)	5.4 (4.8, 5.9)	0.01
Average number of days open	6.7 (6.6, 6.8)	6.6 (6.5, 6.7)	0.04
Average number of hours open	14.3 (13.9, 14.8)	14.3 (13.9, 14.8)	0.96

**Table 5** presents analyses on the availability of ORS and zinc at the outlet on the day of the survey. The proportion of outlets stocking both ORS and zinc was 55% (95% CI = 51%, 59%) in 2014 and 60% (95% CI = 56%, 64%) in 2016. The proportion of outlets stocking any form of ORS (either individually packaged sachets or co-packs) was 75% (95% CI = 71%, 78%) in 2014 and 73% (95% CI = 69%, 77%) in 2016. For any form of zinc (either individually packaged zinc tablets or syrups or co-packs), the proportion was 62% (95% CI = 58%, 66%) in 2014 and 70% (95% CI = 67%, 74%) in 2016. After adjusting for outlet characteristics, specifically the outlet type, region, location in a rural area, the number of staff working at the outlet, the number of years the outlet has been operating, and the number of days and hours the outlet is open, we found a significant association between the survey period and having zinc in-stock. The odds of having any zinc in-stock was 1.5 (95% CI = 1.14, 1.97) times higher in 2016 than in 2014. The association was also significant for zinc tablets (*P* = 0.01) and zinc syrups (*P* < 0.01). We did not find that stocking both ORS and zinc, the co-pack, any ORS, or individually packaged ORS were significantly different in 2016 than in 2014.

**Table 5:** Change in ORS and zinc stocking between 2014 and 2016

	Proportion of outlets stocking (95% CI)		cOR (95% CI)	aOR* (95% CI)	*P*-value
	**2014**	**2016**			
	N = 538	N = 588			
Both ORS and zinc†	0.55 (0.51, 0.59)	0.60 (0.56, 0.64)	1.24 (1.01, 1.54)	1.22 (0.95, 1.55)	0.12
Any ORS‡	0.75 (0.71, 0.78)	0.73 (0.69, 0.77)	0.93 (0.71, 1.21)	0.86 (0.65, 1.13)	0.28
Any zinc§	0.62 (0.58, 0.66)	0.70 (0.67, 0.74)	1.45 (1.17, 1.81)	1.50 (1.14, 1.97)	<0.01
Co-pack	0.07 (0.05, 0.10)	0.06 (0.05, 0.09)	0.93 (0.55, 1.58)	1.03 (0.54, 1.96)	0.92
ORS sachet	0.74 (0.71, 0.78)	0.72 (0.68, 0.76)	0.89 (0.68, 1.16)	0.80 (0.60, 1.06)	0.12
Zinc tablet	0.60 (0.56, 0.64)	0.68 (0.65, 0.72)	1.44 (1.15, 1.79)	1.47 (1.11, 1.93)	0.01
Zinc syrup	0.04 (0.02, 0.06)	0.07 (0.05, 0.09)	1.87 (1.12, 3.15)	2.67 (1.42, 5.02)	<0.01

cOR – crude odds ratio, aOR – adjusted odds ratio, ORS – oral rehydration salts

*Model was adjusted for region, outlet type, rural location, number of workers at the outlet, number of years operating, number of working days in a week, and number of working hours in a day.

†Both ORS and zinc is defined as stocking on the day of the interview either the co-pack or ORS sachets and zinc tablets or syrups.

‡Any ORS is defined as stocking on the day of the interview either the co-pack or ORS sachets.

§Any zinc is defined as stocking on the day of the interview either the co-pack or zinc tablets or syrups.

**Table 6** presents the mean price of ORS and zinc (in USD) after adjusting for inflation. In 2014, we found the mean price of a single ORS sachet was US$0.18 (95% CI = 0.18, 0.19), and in 2016, the mean price was US$0.17 (95% CI = 0.16, 0.18). For zinc, we found mean price of 10 zinc tablets was US$1.00 (95% CI = 0.90, 1.10) in 2014 and US$0.82 (95% CI = 0.74, 0.91) in 2016. Among outlets with both individually packaged ORS sachets and zinc tablets in-stock, the mean price of purchasing a full treatment course (two ORS sachets and 10 zinc tablets) was US$1.36 (95% CI = 1.27, 1.45) in 2014 and US$1.21 (95% CI = 1.11, 1.31) in 2016. The mean price of co-packaged ORS and zinc was US$1.02 (95% CI = 0.82, 1.22) in 2014 and US$0.75 (95% CI = 0.55, 0.95) in 2016, though we found very few co-packs in private outlets. After adjusting for outlet characteristics, we found a statistically significant decrease in the average price of ORS (*P* < 0.01), zinc (*P* < 0.01), and the full treatment (*P* < 0.01) between 2014 and 2016. We did not find a statistically significant difference in the price of the co-pack between 2014 and 2016.

**Table 6:** Change in ORS and zinc retail price (in US$) between 2014 and 2016

	Mean price (95% CI)	Crude mean price difference (95% CI)	Adjusted mean price difference* (95% CI)	*P*-value
**Co-pack:**				
2014 (n = 36)	1.02 (0.82, 1.22)	Ref	Ref	
2016 (n = 39)	0.75 (0.55, 0.95)	-0.28 (-0.57, 0.02)	-0.23 (-0.53, 0.08)	0.14
ORS sachet				
2014 (n = 435)	0.18 (0.18, 0.19)	Ref	Ref	
2016 (n = 462)	0.17 (0.16, 0.18)	-0.01 (-0.02, 0)	-0.02 (-0.03, -0.01)	<0.01
**10 zinc tablets:**				
2014 (n = 340)	1.00 (0.90, 1.10)	Ref	Ref	
2016 (n = 420)	0.82 (0.74, 0.91)	-0.18 (-0.30, -0.06)	-0.20 (-0.31, -0.09)	<0.01
**Full treatment:†**				
2014 (n = 270)	1.36 (1.27, 1.45)	Ref	Ref	
2016 (n = 319)	1.21 (1.11, 1.31)	-0.15 (-0.26, -0.03)	-0.19 (-0.31, -0.06)	<0.01

cOR – crude odds ratio, CI – confidence interval, aOR – adjusted odds ratio, ORS – oral rehydration salts

* Model was adjusted for region, outlet type, rural location, number of workers at the outlet, number of years operating, number of working days in a week, and number of working hours in a day

† Full treatment is defined as 2 ORS sachets and 10 zinc tablets. This is equivalent to a co-pack.

## DISCUSSION

Coverage of the combined use of ORS and zinc has increased rapidly between 2011 and 2016. Children with diarrhoea seeking care in both the public and private sectors are much more likely to receive ORS and zinc in 2016 than they were in 2011, with over half of diarrhoea episodes in the public sector receiving ORS and zinc and about a third in the private sector. Review of documents from partners active in the DPCC suggest that the coordinated and sustained efforts by the DPCC, including the Government of Uganda, health development partners, and private pharmaceutical companies helped increase ORS and zinc coverage between 2011 and 2016. The DPCC was the largest coordinating body focusing on ORS and zinc and had more than 10 organizations actively participating. Therefore, activities by members of the DPCC are likely to be representative of efforts to improve ORS and zinc use in the country.

While coverage of ORS and zinc has been published in Uganda’s DHS reports, this study went further by estimating coverage changes among patients with diarrhoea episodes seeking care in the public and private sectors. This study also included an extensive review of documents from the government and groups active in the DPCC and analysis of private medicine outlet surveys to provide additional context that may have contributed to the rapid coverage changes found in the DHS.

Our evaluation suggests that the DPCC activities in the public sector, particularly the switch to co-packaged ORS and zinc and the widespread dissemination of this information to NMS, district health offices, and facilities, likely played an important role in the increase of both ORS and zinc. In 2016, 77% of patients with diarrhoea episodes seeking care from public sources were receiving ORS and 53% were receiving both ORS and zinc. Though NMS was only distributing the co-pack by 2016, there still appears to be some cases receiving ORS without zinc, and more importantly, a substantial proportion of patients are not receiving ORS at all. Tremendous progress has been made in Uganda’s public sector to improve use of ORS and zinc though further work is still needed to improve coverage among the remaining population not receiving the appropriate treatments.

In the private sector, there has also been significant improvement in the use of both ORS and zinc for treatment of diarrhoea. In 2016, about a third of diarrhoea cases were receiving both ORS and zinc while in 2011, nearly none were. Among private medicine outlets, ORS availability has remained high between 2014 and 2016, and zinc availability has increased. The volume of zinc sold through private sector channels also substantially increased between 2012 and 2015. In the private sector, there was also an increase in the number of products in the market, and we believe the increased competition led to lower prices of ORS and zinc. The price however is still higher than the recommended retail price of UGX 1500 (USD $0.58) promoted by the DPCC and the Government of Uganda. Notably, the average retail price of a co-pack was much less than a full treatment course via individually packaged ORS and zinc. However, the co-pack was launched toward the end of the review period and was only available in 6% of private medicine outlets. Further uptake of co-packs in private medicine outlets may help increase affordability. Also of note, though the DPCC partners conducted several activities to increase the availability and decrease the price of ORS and zinc in the private sector, the DPCC partners did not donate free or subsidized products, which would have compromised long-term sustainability. Instead, DPCC partners supported local distributors and manufacturers to register and launch new, more affordable, and patient-friendly ORS and zinc products.

Our study design was a retrospective analysis of pre-program and post-program DHS data, and therefore, we are unable to conclusively attribute ORS and zinc coverage changes to the DPCC activities. Although, the WHO and UNICEF recommended zinc as an adjunct therapy to ORS in 2004; by 2011, ORS and zinc coverage had only reached 1% in Uganda. The rapid increase in ORS and zinc coverage from 1% to 30% over the five-year period since 2011 represents a dramatic change, which our study suggests is likely the efforts of the DPCC. The study was also not designed to evaluate any specific intervention of the program so we are unable to quantify the relative contribution of any specific intervention to changes in coverage. We suggest future national program evaluations incorporate impact evaluation studies to provide evidence of the impact of specific interventions.

Results from the private medicine outlet surveys were also limited in several ways. First, data collection was limited to 2014 and 2016 though efforts by the DPCC began at the end of 2011. A comparable survey conducted in 2011 would have provided a more rigorous evaluation of the changes occurring in the private sector due to the activities by the DPCC and its partners. We found one study conducted by Research World International and UHMG that found between 44% to 49% drug shops were stocking UHMG’s Zinkid in 2011 [43]. Zinkid was the predominant zinc product in Uganda at the time and so the findings likely represent the availability of zinc at the time. Also, the study found that the price of Zinkid (adjusting for inflation) was US$1.41. Thus, the results from the outlet surveys likely underestimate the impact of the DPCC’s activities in improving the availability and affordability of ORS and zinc. Like with coverage results, we also cannot attribute changes in ORS and zinc availability and price to the DPCC’s activities. While we try to control for differences in the outlet sample populations in 2014 and 2016, there may be other confounding factors unaccounted for in the analysis that are contributing to the changes. Data collection for the surveys also occurred in different months of the year which may pose comparability challenges. Incidence of diarrhoea is seasonal, often coinciding with rain, so the availability of ORS and zinc may also vary throughout the year [44-46]. Although the data was collected at different times of the year, both periods of data collection occurred during the rainy seasons of Uganda and had similar rainfall during the months of data collection [47].

Lastly, the authors of this evaluation were members of the DPCC and had donor-funding to support the DPCC in its efforts to improve ORS and zinc coverage in Uganda. We acknowledge that there may be inherent bias to publish positive results in this study; however, the coverage results used the DHS surveys, which were independently collected using standardized methods. This evaluation may have benefited by having an independent researcher as part of the study team to conduct the document review and data analysis. Funding was not available to hire an independent evaluator to be included in this study.

## CONCLUSION

Use of combined ORS and zinc for treatment of diarrhoea in children under five significantly increased in Uganda during the program period. The range of activities conducted by the various members of the DPCC likely contributed to the increase in the use of combined ORS and zinc.

## Additional material

Online Supplementary Document
