# Supplementary material for: A retrospective mixed-methods evaluation of a national ORS and zinc scale-up program in Uganda between 2011 and 2016
Source: J Glob Health. 2019 Jun 15;9(1):010504. doi: 10.7189/jogh.09.010504 (PMC6571111; doi:10.7189/jogh.09.010504)
Supplement: Online Supplementary Document [file jogh-09-010504-s001.pdf]

J Glob Health 2019; 9: 010504

Online Supplementary Document

Lam et al. A retrospective mixed-methods evaluation of a national ORS and zinc scale-up program in Uganda between 2011 and 2016

Contents

Table S1. Diarrhoea and Pneumonia Coordinating Committee participants 2

Table S2. Documents reviewed 3

Table S1. Diarrhoea and Pneumonia Coordinating Committee participants

Department / Organization / Company	Type (Government, Health Development Partner, Private Pharmaceutical, Other)
Ministry of Health – Child Health Department	Government
Ministry of Health – Health Promotion Department	Government
Ministry of Health – Nursing Department	Government
National Drug Authority (NDA)	Government
Uganda Pediatric Association (UPA)	National Professional Association
Mulago National Referral Hospital	Public Institution
Makerere University College of Health Sciences, School of Medicine (Child Health and Development Center)	Public Institution
World Health Organization (WHO)	Health Development Partner
United Nations Children's Fund (UNICEF)	Health Development Partner
United States Agency for International Development (USAID)	Health Development Partner
Clinton Health Access Initiative (CHAI)	Health Development Partner
Abt Associates / Strengthening Health Outcomes through the Private Sector (SHOPS)	Health Development Partner
Uganda Health Marketing Group (UHMG)	Health Development Partner
Programme for Accessible Health Communication and Education (PACE)	Health Development Partner
Malaria Consortium	Health Development Partner
AMREF	Health Development Partner
World Vision	Health Development Partner
WaterAid	Health Development Partner
BRAC	Health Development Partner
Plan International	Health Development Partner
Uganda Water and Sanitation NGO Network (UWASNET)	Health Development Partner
FHI360 – Communicating for Healthy Communities (CHC)	Health Development Partner
International Rescue Committee (IRC)	Health Development Partner
Kampala Pharmaceutical Industries (KPI)	Private Pharmaceutical Company
Medipharm	Private Pharmaceutical Company
Gittoes Pharmaceutical	Private Pharmaceutical Company
Phillips Pharmaceuticals /Star Pharmaceuticals	Private Pharmaceutical Company

Table S2. Documents reviewed

Type	Title	Authors	Year	Published (Yes/No)	Main information used in the document review
National guidelines	Uganda Clinical Guidelines: National Guidelines on Management of Common Conditions	Ministry of Health	2010	Yes	Zinc included in the national clinical guidelines
National guidelines	Essential Medicines and Health Supplies List for Uganda.	Ministry of Health	2012	Yes	Zinc included in the EML
National strategy document	Child Survival Strategy for Uganda 2009/10-2014/15	Uganda Ministry of Health	2010	Yes	ORS and zinc national coverage target
National strategy document	Reproductive Maternal Neonatal Child Health Sharpened Plan for Uganda	Uganda Ministry of Health	2013	Yes	ORS and zinc national coverage target
National strategy document	Scaling up Approaches to Protect, Prevent and Treat Diarrhea and Pneumonia in Uganda	Uganda Ministry of Health	2014	Yes	ORS and zinc national coverage target; ORS-zinc scale up partner mapping
Report	Uganda Demographic and Health Survey, 2011	UBOS and Macro International Inc.	2012	Yes	ORS and zinc national and regional coverage in 2011; radio penetration rate and audience measurement
Report	Uganda Demographic and Health Survey, 2016	UBOS and Macro International Inc.	2017	Yes	ORS and zinc national and regional coverage in 2016
Report	Assessment on the management of diarrhea, malaria and pneumonia in children under five	Clinton Health Access Initiative	2016	No	ORS and zinc availability in public health facilities in 2016
Report	Reducing Diarrhea Mortality and Morbidity in Uganda: Scaling-up Zinc/ORS, Progress Report: October 2012 – June 2013	Clinton Health Access Initiative	2013	No	Import prices of zinc products registrered during the program lifetime compared to those already registered before the program onset.
Report	Reducing Diarrhea Mortality and Morbidity in Uganda: Scaling-up Zinc/ORS, Progress Report: December 2013 – May 2014	Clinton Health Access Initiative	2014	No	Scale and scope of the supply and demand activities at pharmaceutical wholesale level in 2014; nature of the partnerships between CHAI and two pharmaceutical distributors in Uganda.
Report	Reducing Diarrhea Mortality and Morbidity in Uganda: Scaling-up Zinc/ORS, Final Narrative Report:	Clinton Health Access Initiative	2016	No	Scale and scope of radio campaign in 2014 and 2015

	December 2015 – May 2016				
Report	Strengthening Health Outcomes through the Private Sector Project: Final Report 2009–2016	SHOPS Project	2016	Yes	SHOPS training of private health providers between 2013 and 2014
Report	Annual Report 2013-2014.	Uganda Health marketing Group (UHMG)	2014	Yes	BCC campaign led by UHMG in 2013
Report	Performance Evaluation of USAID/Uganda AFFORD – Health Marketing Initiative Project Evaluation Improving the Lives of Ugandans	Ankomah A., Spicehandler J., Kibuuka F., Mobley S.	2013	Yes	Key info about UHMG's zinc product (launch date, subsidy, funding source)
Report	Lessons from Uganda on Integrating the Integrated Community Case Management Model (iCCM)	Maternal and Child Survival Program.	2015	Yes	ICCM partner list
Report	Marketing Zinc for Childhood Diarrhea Treatment: Results from household and provider surveys in Benin and Uganda	Abt Associates Inc.	2012	Yes	Caregivers' preferred diarrhea treaments in 2012
Report	Pharmaceutical Sector Profile Uganda	United Nations Industrial Development Organization,	2010	Yes	Key facts about the pharmaceutical industry in Uganda; name of the ORS manufacturer in 2010
Report	Supply Chain System for Community Health Programs in Uganda: a Situation Analysis	Uganda Ministry of Health and USAID/Uganda Health Supply Chain Program	2016	Yes	Integration of iCCM supply chain into the national supply chain and transfer of the distribution responsibility to the National Medical Store
Minutes of meetings	Minutes from private supplier forum meetings, July 2013	Clinton Heath Access Initiative	2013	No	Role and scope of the private suppliers forum; activities of the key participating companies
Minutes of meetings	Minutes from private supplier forum meetings, January 2014	Clinton Heath Access Initiative	2014	No	Role and scope of the private suppliers forum; activities of the key participating companies
Minutes of meetings	Minutes from private supplier forum meetings, May 2014	Clinton Heath Access Initiative	2014	No	Role and scope of the private suppliers forum; activities of the key participating companies
Minutes of meetings	Minutes from private supplier forum meetings, April 2015	Clinton Heath Access Initiative	2015	No	Role and scope of the private suppliers forum; activities of the key participating companies
Minutes of meetings	Minutes from private supplier forum meetings, November 2015	Clinton Heath Access Initiative	2015	No	Role and scope of the private suppliers forum; activities of the key participating companies

Minutes of meetings	Meeting minutes of the 2nd Diarrhea & Pneumnia Coordination Committee meeting on 16 Oct 2012	Uganda Ministry of Health and Diarrhea & Pneumnia Coordination Committee	2012	No	Activities of the DPCC partners
Minutes of meetings	Meeting minutes of the 3rd Diarrhea & Pneumnia Coordination Committee meeting on 5 Dec 2012	Uganda Ministry of Health and Diarrhea & Pneumnia Coordination Committee	2012	No	Activities of the DPCC partners
Minutes of meetings	Meeting minutes of the 4th Diarrhea & Pneumnia Coordination Committee meeting on 20 May 2013	Uganda Ministry of Health and Diarrhea & Pneumnia Coordination Committee	2013	No	Activities of the DPCC partners; Zinc over-the-counter status approval; NMS co-pack procurement
Presentation	ICCM in Uganda: background and process	Dr. Henry Katamba, Ministry of Health	2013	Yes	Scale and scope of ICCM program in Uganda
Presentation	Improving access to essential medicines in the private sector	Clinton Health Access Initiative	2014	No	Description of the private pharmaceutical supply chain in Uganda: number of first line importers, Kampala-based and regional pharmacy wholesalers
Presentation	Introducing Recommended Retail Prices for ACTs and Zinc-ORS in the Private Sector	Clinton Heath Access Initiative	2014	No	Recommended Retail price level and date of introduction
